# Association of Physical Activity and Risk of Stroke: A Prospective Cohort Study of Chinese Middle‐aged and Older Adults

**DOI:** 10.1002/brb3.71316

**Published:** 2026-03-31

**Authors:** Hao Jia, Yuhong Chen, Lin Dou, Baoqi Zeng

**Affiliations:** ^1^ Drug Clinical Trial Institution, Tianjin Fifth Central Hospital Peking University Binhai Hospital Tianjin China; ^2^ Department of Orthopedics, Tianjin Fifth Central Hospital Peking University Binhai Hospital Tianjin China; ^3^ Department of Emergency & Emergency Medicine Research Institute, Tianjin Fifth Central Hospital Peking University Binhai Hospital Tianjin China; ^4^ Central Laboratory, Tianjin Fifth Central Hospital Peking University Binhai Hospital Tianjin China; ^5^ Department of Epidemiology and Biostatistics, School of Public Health Peking University Beijing China

**Keywords:** aged, CHARLS, Cohort, physical activity, stroke

## Abstract

**Purposes:**

To investigate the relationship between different physical activity (PA) patterns and stroke incidence among middle‐aged and elderly populations in China.

**Methods:**

Data were drawn from the China Health and Retirement Longitudinal Study (CHARLS), a nationally representative prospective cohort encompassing 2011 to 2020. PA was calculated based on the International Physical Activity Questionnaire. Different patterns of PA included moderate‐to‐vigorous PA (MVPA, ≥ 150 min/wk vs. < 150 min/wk), vigorous PA (VPA, ≥ 75 min/wk vs. < 75 min/wk), moderate PA (MPA, ≥ 150 min/wk vs. < 150 min/wk), light PA (LPA, ≥ 300 min/wk vs. < 300 min/wk), and total PA (TPA, ≥ 600 metabolic equivalent of task [MET]‐min/wk vs. < 600 MET‐min/wk). Cox proportional hazards models evaluated stroke risk associations, while restricted cubic splines (RCS) characterized TPA dose‐response effects.

**Results:**

There were 5090 participants in total (mean age, 59.23 [standard deviation, 9.43] years; 54.5% were female), and 378 (7.4%) incident stroke cases were documented at a 9‐year follow‐up. Achieving the World Health Organization (WHO) guideline of ≥150 min/wk MVPA was associated with a 24% lower stroke risk (adjusted hazard ratio [HR] = 0.77, 95% confidence interval [CI] = 0.62–0.96, *p* = 0.019). No significant association was observed between VPA (HR = 0.79, 95% CI 0.63–1.01), MPA (HR = 0.82, 95% CI = 0.67–1.01), LPA (HR = 0.86, 95% CI = 0.70–1.07), or TPA (HR = 0.84, 95% CI = 0.65–1.08) and stroke risk. Additionally, RCS analysis demonstrated a non‐significant dose‐response relationship between TPA and stroke risk.

**Conclusion:**

This study validates WHO's MVPA guidelines (≥ 150 min/wk) for stroke prevention in Chinese elders. However, the predominantly self‐reported and occupation‐based PA in this cohort highlights the need for future research focusing on objective measurements of leisure‐time PA.

## Graphical abstract

In this 9‐year prospective cohort study of over 5000 middle‐aged and older Chinese adults, we found that achieving the World Health Organization (WHO) guideline of at least 150 min/week of moderate‐to‐vigorous physical activity (MVPA) is associated with a lower risk of stroke. These findings validate current WHO recommendations for stroke prevention in Chinese elders.

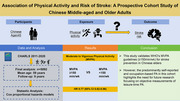

## 1 Introduction

Stroke, an acute focal neurological disorder due to ischemia or hemorrhage, ranks among the leading causes of global death and disability (Shao et al. 2024). Projections indicate a 34% rise in incidence from 2015 to 2035 (McLellan et al. 2024). As a major developing country, China shoulders the world's heaviest stroke burden. China's stroke burden reached 3.94 million incident cases (total prevalent cases: 28.76 million) in 2019, reflecting an 86% prevalence surge since 1990 (Wang et al. 2022). Stroke disproportionately affects middle‐aged and older adults, with prevalence projected to rise amid China's demographic shift (Jiang et al. 2024).

Evidence demonstrates the stroke‐preventive benefits of physical activity (PA) in the general population (McLellan et al. 2024). World Health Organization (WHO) 2020 guidelines recommend adults engage in ≥ 150 min/week of moderate‐ or ≥ 75 min/week of vigorous‐intensity physical activity, or equivalent combinations (Bull et al. 2020). Evidence shows that compared to the inactive, those who engage in ≥ 30 min of moderate‐to‐vigorous physical activity (MVPA) 1–2 times/week have a 16% reduction in stroke risk. Similarly, engaging in MVPA 3–4 times/week and ≥ 5 times/week is associated with 21% and 22% reductions in stroke risk, respectively (McLellan et al. 2024). Evidence indicates enhanced PA benefits in populations with prevalent cardiometabolic risk factors (e.g., hypertension, hyperglycemia) (Stewart et al. 2017; Jeong et al. 2019).

Beyond these direct cardiometabolic benefits, PA contributes to what is termed “motor and cognitive reserve,” a construct describing the brain's inherent resilience to pathology and injury. Premorbid PA is theorized to build this reserve through enhancements in neural plasticity and cerebrovascular health, creating a buffer that may both lower stroke risk and improve recovery outcomes after a stroke (Giustiniani and Quartarone 2024). Empirical evidence supports this dual role. Higher levels of PA are associated with a graded reduction in ischemic stroke incidence (Kyu et al. 2016). Importantly, this pre‐stroke investment appears to pay dividends post‐stroke, as greater premorbid PA is associated with better functional recovery and reduced disability and may lower the risk of serious complications (van Allen et al. 2024; Nozoe et al. 2024; Wang et al. 2025).

Many previous researchers have explored the relationship of PA and stroke risk (McLellan et al. 2024; Jiang et al. 2024; Yu et al. 2018; Yang et al. 2020). However, prospective evidence linking specific PA patterns to stroke risk in Chinese adults remains scarce. The context is unique, with a large rural and aging population often engaged in high volumes of occupational PA. It is unclear whether this predominantly work‐related PA confers protection against stroke comparable to the benefit suggested by guidelines based on leisure‐time activity. This prospective cohort study employed longitudinal data from the China Health and Retirement Longitudinal Study (CHARLS) to explore PA‐stroke associations in Chinese elders, generating epidemiological evidence for targeted prevention strategies.

## 2 Methods

### 2.1 Study Population

Analyses utilized five‐wave longitudinal data (2011–2020) from the nationally representative CHARLS cohort, which employed multistage probability sampling to enroll participants aged ≥45 years across 28 provinces' urban‐rural populations (project details: http://charls.pku.edu.cn/) (Zhao et al. 2014). The protocol received ethical clearance from the Peking University Institutional Review Board (No. IRB00001052‐11015), with written informed consent obtained per Declaration of Helsinki principles.

**FIGURE 1:**
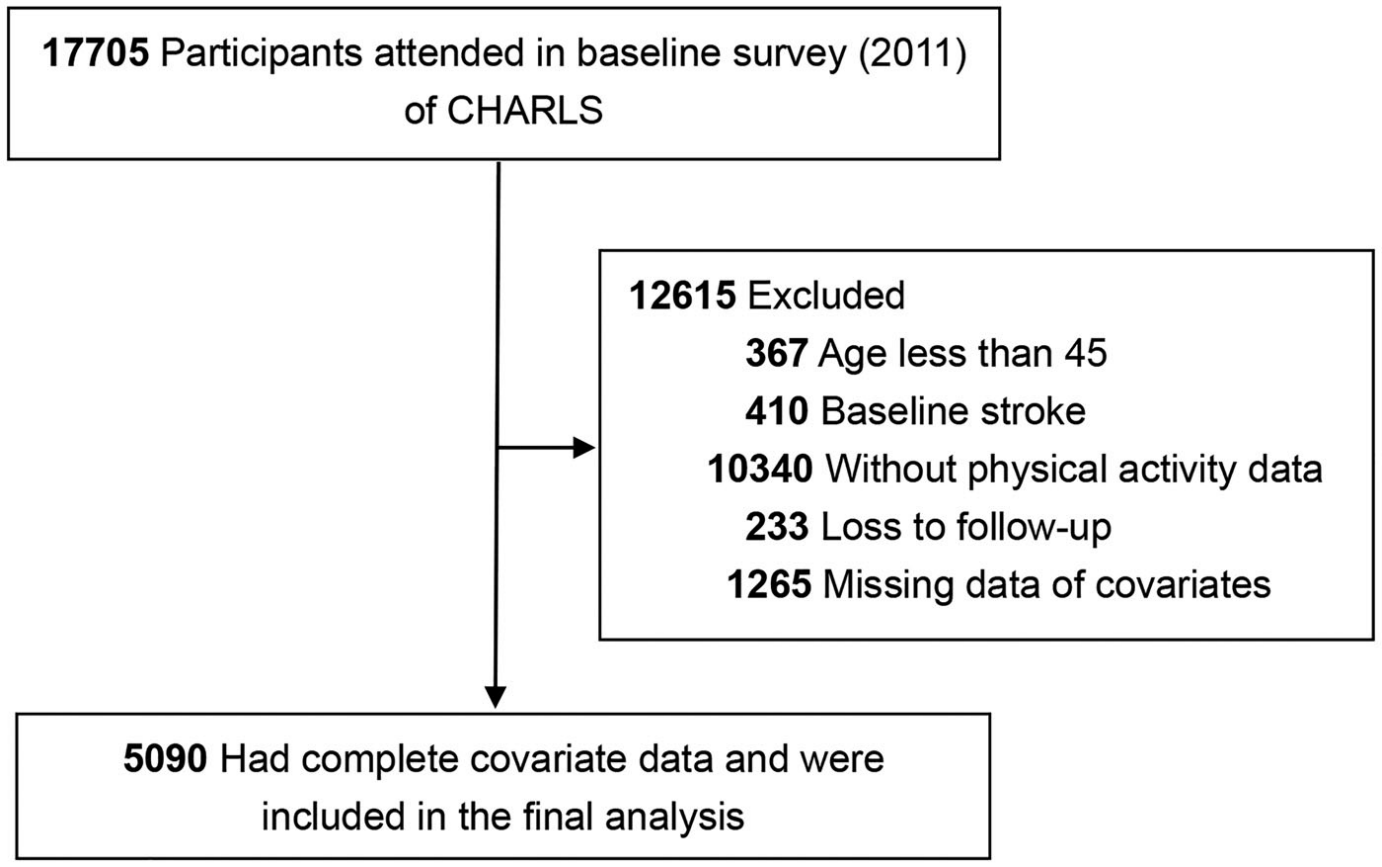
Flow of study participants.

ID was used to integrate the function and health status data from the five waves. Of the 17,705 participants at baseline, we excluded 367 people younger than 45 years, 410 participants with a stroke history at baseline, 10,340 people with no PA data, and 233 individuals with no information of follow‐up. Participants with missing data on key covariates were also excluded (*n* = 1265). Finally, the final analysis comprised 5090 individuals. Figure 1 displays the study's flow chart.

### 2.2 Physical Activity

PA data were obtained through a modified International Physical Activity Questionnaire (IPAQ) assessing weekly engagement (≥10 min/session) across three intensity domains: light‐intensity physical activity (LPA: walking, leisure sports); moderate‐intensity physical activity (MPA: floor mopping, cycling, light‐load carrying, Taijiquan); and vigorous‐intensity physical activity (VPA: heavy‐load lifting, farming, aerobics, fast cycling). Then, the participants were asked, “How many days in a typical week did you perform VPA/MPA/LPA for at least 10 min?” and “How much time did you usually spend performing VPA/MPA/LPA on one of those days (< 30 min or ≥ 30 min, < 2 hrs or ≥ 2 hrs, < 4 hrs or ≥ 4 hrs).” To standardize quantification given CHARLS' temporal data limitations, PA bout durations were imputed using category‐specific medians. This approach was chosen as a pragmatic midpoint estimate within each categorical range to enable quantitative analysis. Values were truncated at < 10 min (assigned 0) in alignment with the IPAQ's definition of a valid 10‐min activity bout and at ≥ 240 min (capped at 240) to minimize the influence of extreme outliers and potential over‐reporting on total volume estimates, a common procedure in epidemiological studies using self‐reported PA data (Jiang et al. 2023; Ding et al. 2022). Weekly duration for each intensity was derived by multiplying the daily bout duration by the weekly frequency. MVPA was calculated as the sum of time spent in MPA and VPA. Total physical activity (TPA) quantification employed metabolic equivalent of task (MET)‐hour/week units, calculated as: TPA = 8.0 × VPA (min/week) + 4.0 × MPA (min/week) + 3.3 × LPA (min/week) (Jiang et al. 2024). Standardized MET coefficients were applied: 3.3 (LPA), 4.0 (MPA), 8.0 (VPA). Weekly duration per intensity derived from daily bout duration × weekly frequency. The WHO has recommended at least 150 min/week of MPA or 75 min/week of VPA. For further health advantages, the WHO has also suggested doing at least 300 min of MVPA every week (Bull et al. 2020). We divided participants into two groups (< 150 min/week and ≥ 150 min/week) depending on the WHO's recommendation. The 2011 baseline questionnaire did not ascertain the purpose (occupational or leisure) of the reported physical activity. This information was, however, collected in a subsequent survey (2015, 2018, and 2020) wave which included questions on activity purpose.

### 2.3 Stroke

Stroke diagnosis was ascertained by asking participants, “Has a doctor ever diagnosed you with having a stroke?” Stroke case ascertainment relied on self‐reported affirmative responses to physician‐diagnosed stroke events. The follow‐up period was chosen as the timeframe that began with baseline (2011) and ended with stroke diagnosis, death, loss to follow‐up, or the latest data update (2020).

### 2.4 Covariates

Covariates were analyzed across three domains (Shao et al. 2024): Sociodemographics included age strata (45–54, 55–64, 65–74, ≥ 75 years), sex (male or female), educational attainment (illiterate, primary, or secondary and above), residence (urban or rural), marital status (married vs. unmarried/widowed/divorced), and body mass index (BMI) categorized as underweight (< 18.5 kg/m2), normal weight (18.5–23.9 kg/m2), overweight (24.0–27.9 kg/m2), or obese (≥ 28 kg/m2) (McLellan et al. 2024). Lifestyle factors comprised current smoking and alcohol consumption status (yes/no) (Wang et al. 2022). Health conditions encompassed hypertension, dyslipidemia, diabetes, cardiopulmonary conditions (such as chronic lung disease, asthma, or cardiac disorders), hepatorenal or gastrointestinal diseases, neurological disorders (including memory‐related or psychiatric conditions), arthritis or rheumatism, and oncological history. All variables were standardized using baseline survey data.

### 2.5 Statistical Analysis

Continuous variables were expressed as means ± standard deviation (SD), while categorical variables were reported as counts and proportions. Intergroup comparisons utilized student's *t*‐test (continuous) and Pearson χ (McLellan et al. 2024) (categorical). Multivariable Cox proportional hazards regression models were used to explore the associations between various patterns of PA and stroke risk. Results reported adjusted hazard ratios (HR) with 95% confidence intervals (CIs), referenced to baseline PA categories. Model 1 incorporated adjustments for core demographic parameters (age, sex, and BMI), followed by Model 2, which subsequently expanded to include residence location, marital status, smoking, and drinking. The fully adjusted model (Model 3) further adjusted for health conditions. The dose‐response associations between TPA (MET‐mins/week) and stroke risk were assessed using Cox proportional hazards models with restricted cubic splines (RCS). The proportional hazards assumption for the RCS model was evaluated using Schoenfeld residuals, and no significant violation was detected (global p > 0.05). The necessity of the non‐linear term was confirmed by a significant likelihood ratio test comparing the RCS model with a linear‐only model (p for non‐linearity = 0.017). Four knots were placed at the fifth, 35th, 65th, and 95th percentiles of the TPA exposure distribution, consistent with the default settings in Stata. The median TPA value was set as the reference point. The median TPA value (5544 MET‐min/week) was set as the reference point to ensure model stability and clinical interpretability within the observed data range. Exploratory subgroup analyses were performed to assess the potential modification of the association between guideline‐based MVPA and stroke risk by pre‐specified demographic and clinical factors: age, sex, BMI, residence, marital status, smoking, drinking, hypertension, diabetes, and dyslipidemia. To evaluate potential selection bias, baseline characteristics were compared between the included analytical sample and those excluded solely for missing PA data. We used Stata 17.0 software in all analyses, with two‐sided P < 0.05 indicating significance.

## 3 Results

This study included 5090 participants (mean age, 59.23 [SD, 9.43] years; 54.5% were female), and 378 (7.4%) incident stroke cases were documented at a 9‐year follow‐up. The differences between the MVPA <150 min/week and MVPA ≥150 min/week groups at the baseline are shown in Table 1. There were 1885 individuals in the low MVPA group (37.0%) and 3205 in the high MVPA group (63.0%), stratified by the WHO guideline. The WHO‐recommended participants were more likely to be younger (57.65 ± 8.41 vs. 61.91 ± 10.43, *p* < 0.001), underweight (6.5% vs. 7.1%, *p* < 0.001), male (48.7% vs. 40.8%, *p* < 0.001), married (90.6% vs. 82.2%, p<.001), living in a rural area (87.6% vs. 72.7%, *p* < 0.001), and have a lower prevalence of several chronic conditions than those with inadequate MVPA. These included hypertension (19.8% vs. 28.5%, *p* < 0.001), dyslipidemia (7.3% vs. 10.7%, *p* < 0.001), diabetes (3.7% vs. 8.2%, *p* < 0.001), and heart problems (9.1% vs. 16.2%, *p* < 0.001).

**TABLE 1:** Baseline characteristics of participants by moderate‐to‐vigorous physical activity.

Characteristics	Level	MVPA < 150	MVPA ≥ 150	*p*‐value*
Participants, No.		1885	3205	
Age, mean (SD)		61.91 (10.43)	57.65 (8.41)	< 0.001
Age group	< 55	505 (26.8%)	1246 (38.9%)	< 0.001
	55–64	675 (35.8%)	1294 (40.4%)	
	65–74	433 (23.0%)	549 (17.1%)	
	≥ 75	272 (14.4%)	116 (3.6%)	
BMI	Underweight	134 (7.1%)	209 (6.5%)	< 0.001
	Normal weight	926 (49.1%)	1796 (56.0%)	
	Overweight	571 (30.3%)	910 (28.4%)	
	Obesity	254 (13.5%)	290 (9.0%)	
Gender	Male	770 (40.8%)	1562 (48.7%)	< 0.001
	Female	1115 (59.2%)	1643 (51.3%)	
Residence	Rural	1371 (72.7%)	2809 (87.6%)	< 0.001
	Urban	514 (27.3%)	396 (12.4%)	
Education	Illiterate	557 (29.5%)	874 (27.3%)	0.130
	Primary education	744 (39.5%)	1345 (42.0%)	
	Secondary or above	584 (31.0%)	986 (30.8%)	
Marital status	Married	1550 (82.2%)	2904 (90.6%)	< 0.001
	Unmarried/divorced/widowed	335 (17.8%)	301 (9.4%)	
Smoke	Yes	638 (33.8%)	1276 (39.8%)	< 0.001
	No	1247 (66.2%)	1929 (60.2%)	
Drink	Yes	491 (26.0%)	1173 (36.6%)	< 0.001
	No	1394 (74.0%)	2032 (63.4%)	< 0.001
Hypertension	Yes	538 (28.5%)	635 (19.8%)	< 0.001
	No	1347 (71.5%)	2570 (80.2%)	
Dyslipidemia	Yes	201 (10.7%)	234 (7.3%)	< 0.001
	No	1684 (89.3%)	2971 (92.7%)	
Diabetes or high blood sugar	Yes	155 (8.2%)	119 (3.7%)	< 0.001
	No	1730 (91.8%)	3086 (96.3%)	
Cancer or malignant tumor	Yes	25 (1.3%)	25 (0.8%)	0.076
	No	1860 (98.7%)	3180 (99.2%)	
Chronic lung diseases	Yes	197 (10.5%)	312 (9.7%)	0.410
	No	1688 (89.5%)	2893 (90.3%)	
Liver disease	Yes	63 (3.3%)	130 (4.1%)	0.220
	No	1822 (96.7%)	3075 (95.9%)	
Heart problems	Yes	306 (16.2%)	293 (9.1%)	< 0.001
	No	1579 (83.8%)	2912 (90.9%)	
Kidney diease	Yes	108 (5.7%)	225 (7.0%)	0.078
	No	1777 (94.3%)	2980 (93.0%)	
Stomach or other digestive disease	Yes	389 (20.6%)	813 (25.4%)	< 0.001
	No	1496 (79.4%)	2392 (74.6%)	
Emotional, nervous, or psychiatric problems	Yes	19 (1.0%)	29 (0.9%)	0.760
	No	1866 (99.0%)	3176 (99.1%)	
Memory‐related disease	Yes	25 (1.3%)	29 (0.9%)	0.160
	No	1860 (98.7%)	3176 (99.1%)	
Arthritis or rheumatism	Yes	600 (31.8%)	1161 (36.2%)	0.002
	No	1285 (68.2%)	2044 (63.8%)	
Asthma	Yes	84 (4.5%)	111 (3.5%)	0.082
	No	1801 (95.5%)	3094 (96.5%)	

*p‐values are calculated by one‐way analysis of variance for continuous variables and the *x* ^2^ test for categorical variables.

Abbreviation: MVPA, moderate‐to‐vigorous physical activity.

We compared the baseline characteristics of the included participants (*n* = 5090) with those excluded solely for missing PA data (*n* = 7055). The two groups were comparable across most measured covariates. Statistically significant differences were observed only for sex (54.2% vs. 51.3% female) and current smoking status (37.6% vs. 41.5%).

Compared with participants with MVPA less than 150 min/week, those with MVPA ≥ 150 min/week were related to a lower risk of stroke in Model 1 (HR = 0.76, 95% CI = 0.61–0.93, *p* = 0.009), Model 2 (HR = 0.76, 95% CI = 0.62–0.95, *p* = 0.014), and Model 3 (HR = 0.77, 95% CI = 0.62–0.96, *p* = 0.019) (Table 2). We also evaluated other PA patterns' association with stroke risk. Compared to individuals with VPA less than 75 min/week, those with VPA ≥ 75 min/week were not associated with incident stroke (HR 0.79, 95% CI = 0.63–1.01, *p* = 0.058). Additionally, compared with MPA < 150 min/week, a higher MPA level was not associated with stroke risk (HR = 0.82, 95% CI = 0.67–1.01, p = 0.064). Similarly, no significant difference in stroke risk was observed between participants with lower LPA (< 300 min/week) and those with higher LPA (≥300 min/week) (HR = 0.86, 95% CI = 0.70–1.07, *p* = 0.171). For TPA, participants with ≥ 600 MET‐min/week had a similar risk of stroke as those with < 600 MET‐min/week (HR = 0.84, 95% CI = 0.65–1.08, *p* = 0.176).

**TABLE 2:** Hazard Ratios of Associations of Physical Activity and Incident stroke.

Exposures	Event	Participants	Model 1		Model 2		Model 3	
	n	N	HR (95% CI)	p	HR (95% CI)	p	HR (95% CI)	p
MVPA								
< 150 min/week	165	1885	1.00 (ref.)		1.00 (ref.)		1.00 (ref.)	
≥ 150 min/week	213	3205	0.76 (0.61, 0.93)	**0.009**	0.76 (0.62, 0.95)	**0.014**	0.77 (0.62, 0.96)	**0.019**
VPA								
< 75 min/week	264	3268	1.00 (ref.)		1.00 (ref.)		1.00 (ref.)	
≥ 75 min/week	111	1804	0.78 (0.62, 0.98)	**0.035**	0.79 (0.63, 1.00)	0.054	0.79 (0.63, 1.01)	0.058
MPA								
< 150 min/week	195	2386	1.00 (ref.)		1.00 (ref.)		1.00 (ref.)	
≥ 150 min/week	178	2676	0.81 (0.66, 1.00)	**0.047**	0.82 (0.67, 1.01)	0.066	0.82 (0.67, 1.01)	0.064
LPA								
< 300 min/week	195	2386	1.00 (ref.)		1.00 (ref.)		1.00 (ref.)	
≥ 300 min/week	178	2676	0.85 (0.70, 1.06)	0.148	0.86 (0.70, 1.06)	0.147	0.86 (0.70, 1.07)	0.171
TPA								
MET < 600	75	899	1.00 (ref.)		1.00 (ref.)		1.00 (ref.)	
MET ≥ 600	303	4191	0.83 (0.64, 1.07)	0.146	0.83 (0.64, 1.08)	0.162	0.84 (0.65, 1.08)	0.176

Model 1 adjusted for age, sex, and body mass index. Model 2 additionally adjusted residence location, marital status, smoking and drinking. Model 3 further adjusted for hypertension, dyslipidemia, diabetes or high blood sugar, cancer, chronic lung disease, liver disease, asthma, heart problems, kidney disease, memory‐related diseases, stomach or other digestive diseases, emotional, nervous, or psychiatric problems, arthritis, or rheumatism.

Abbreviations: LPA, light physical activity; MET, metabolic equivalent; MVPA, moderate‐to‐vigorous physical activity; TPA, total physical activity.

**FIGURE 2:**
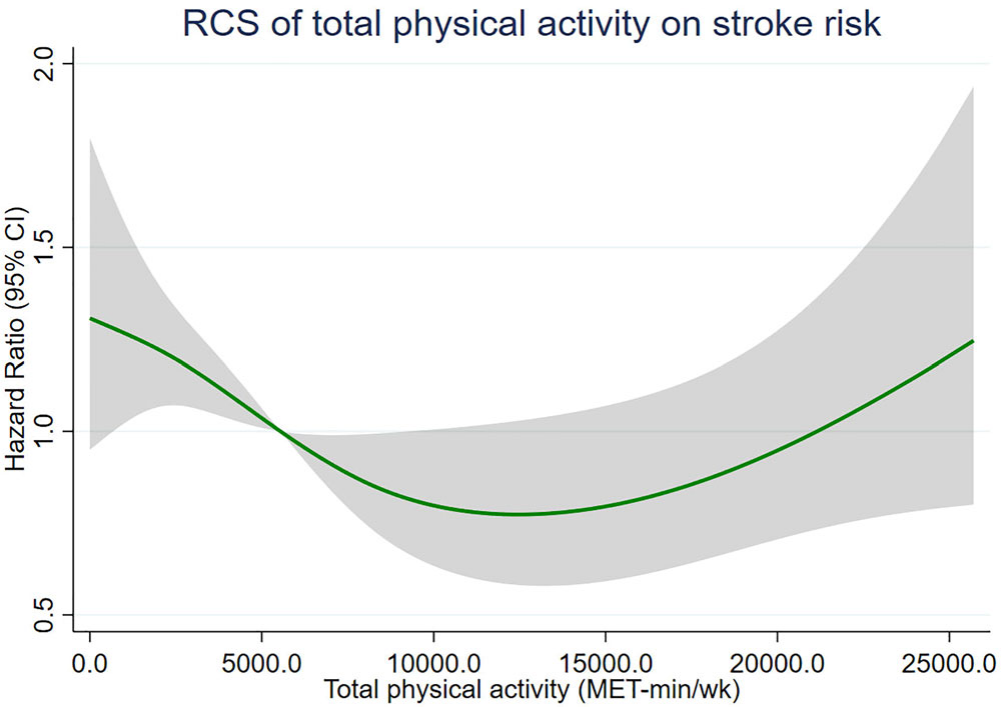
Dose‐response association between TPA and stroke risk. The solid curve represents the adjusted hazard ratio (HR), and the shaded area represents the 95% confidence interval, derived from a restricted cubic spline Cox proportional hazards model. Four knots were placed at the 5th, 35th, 65th, and 95th percentiles of the TPA distribution, and the median value (5544 MET‐min/week) was set as the reference. The dashed horizontal line indicates the null effect (HR = 1).

We also investigated the dose‐response association between TPA (MET‐min/week) and incident stroke. However, the dose‐response analysis showed no significant correlation between TPA and stroke in this study (p = 0.091) (Figure 2). However, the shape of the dose‐response curve was non‐linear (p for non‐linearity = 0.017). In exploratory subgroup analyses, no statistically significant interaction effects were observed between MVPA and any of the pre‐specified factors, including age, sex, BMI, residence location, marital status, smoking, drinking, hypertension, diabetes or high blood sugar, and dyslipidemia (all p for interaction > 0.05).

**FIGURE 3:**
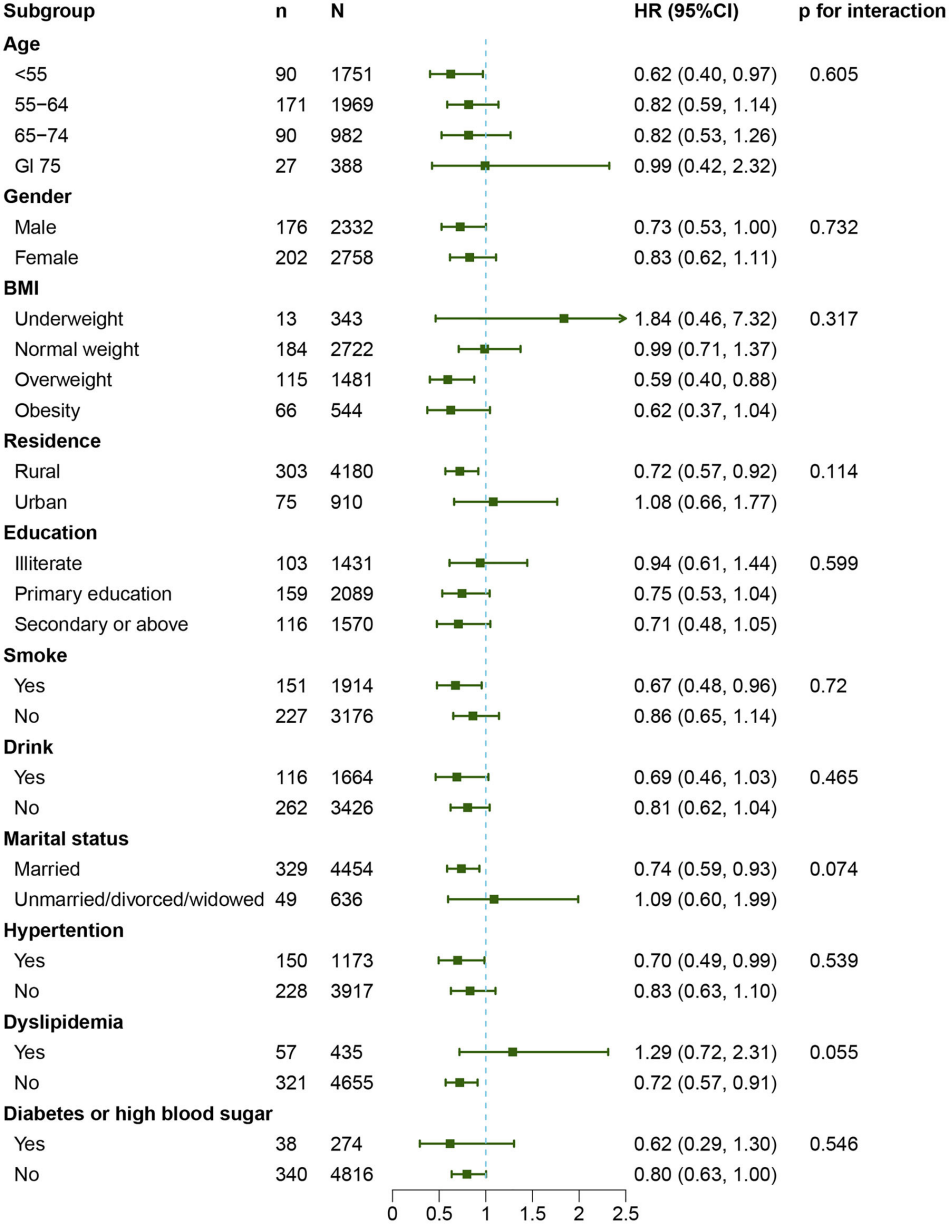
Subgroup analyses of the association between MVPA and stroke.

The association of TPA and stroke was observed among participants who were under 55 (HR = 0.62, 95% CI = 0.40–0.97, p = 0.036), overweight (HR = 0.59, 95% CI = 0.40–0.88, p = 0.009), living in a rural area (HR = 0.72, 95% CI = 0.57–0.92, p = 0.008), smoking (HR = 0.67, 95% CI = 0.48–0.96, p = 0.027), married (HR = 0.74, 95% CI = 0.59–0.93, p = 0.010), with hypertension (HR = 0.70, 95% CI = 0.49–0.99, p = 0.041), and without dyslipidemia (HR = 0.72, 95% CI = 0.57–0.91, p = 0.006). However, no significant interaction effect was observed among all those subgroups. Figure 3 shows the results of the subgroup analyses.

## 4 Discussion

This prospective cohort study enrolled 5090 middle‐aged and older adults and examined the associations between different patterns of PA and risk of stroke at a 9‐year follow‐up. This is the first prospective longitudinal study that explores the relationship between PA and incident stroke in Chinese adults. This study indicated that higher levels of MVPA were significantly associated with a lower risk of stroke, supporting prior research (Jeong et al. 2017; Pinckard et al. 2019). In contrast, the effects of LPA, MPA, VPA, and TPA were insignificant. Dose‐response analysis found no TPA‐stroke correlation (*p* = 0.091). Subgroup analyses indicated an association between PA and stroke among participants under 55; overweight; living in a rural area; smoking; married; with hypertension; and without dyslipidemia.

No association was observed between VPA (≥ 10 min/week vs. < 10 min/week), MPA (≥ 10 min/week vs. < 10 min/week), and TPA (≥ 600 MET‐min/week vs. < 600 MET‐min/week) and the risk of stroke. In contrast, some previous studies found associations between these PA patterns and stroke (Jiang et al. 2024; Yang et al. 2020; Kubota et al. 2017). Jiang et al. (2024) and Yang et al. (2020) suggested that high frequency or long duration of MPA and VPA was associated with a lower risk of stroke. Furthermore, Jiang found that higher TPA was linked to a lower risk of stroke (Jiang et al. 2024). However, most of these studies are cross‐sectional studies or do not involve Chinese populations. Therefore, more large prospective cohort studies on Chinese populations are needed in the future to evaluate the association between different patterns of VPA, TPA, and MPA and stroke risk.

Regarding LPA, no significant correlation was found between LPA and the risk of stroke. Our findings are in line with those of several prior studies (Jiang et al. 2024; Yang et al. 2020; Lacroix et al. 2019). LPA is not advised for stroke prevention in middle‐aged and older populations, as its minimal energy expenditure provides inadequate stimulation for enhancing cardiopulmonary function, circulatory efficiency, or metabolic regulation (Zheng et al. 2023; Niemelä et al. 2024).

Multiple contributors are linked to cerebrovascular events, such as advancing age, excessive body weight, unbalanced nutrition, physical inactivity, and tobacco use (O'Donnell et al. 2016). The World Stroke Organization (WSO) highlights hypertension, elevated BMI, and diabetes as key determinants of cerebrovascular accidents (Feigin et al. 2022), with data indicating that elevations in systolic/diastolic blood pressure by 3.0/2.3 mmHg correlate with a 24% higher cerebrovascular accident rate (Poirier et al. 2006). Engaging in Routine PA can reduce blood pressure by 5 mmHg overall and improve lipid profiles and also improve endothelial function, which enhances vasodilation and vasomotor function (Jiang et al. 2024; García‐Cabo 2020). Our subgroup analyses showed that guideline‐based MVPA demonstrated reduced stroke risk in patients with hypertension but not in normotensive patients.

Our investigation revealed that 82.7% of participants resided in non‐urban regions; these individuals routinely performed strenuous agricultural tasks, including crop cultivation and earth excavation on a near‐daily basis. Evidence suggests regular involvement in agrarian activities within environmentally sustainable settings significantly decreases cardiovascular disease/stroke occurrence (Sun et al. 2023). Li et al.’s study demonstrated countryside dwellers undertaking ≥ 40 weekly hours of MVPA exhibited 45% reduced cardiovascular disease/stroke mortality risk compared to physically inactive counterparts (2024).

The 2011 baseline survey contained no purposeful information regarding physical activity. However, according to the 2020 survey, there was a 42.6% job demand for MPA (1092/2561) and a 69.1% job demand for VPA (1160/1679). Furthermore, MPA had a 27.3% entertainment or excise purpose (375/2561), whereas VPA had a 22.3% entertainment or excise purpose (375/1679). The majority of participants with MVPA ≥ 150 min/week were likely doing so for work‐related reasons. This context is important when interpreting the associations, as emerging evidence describes a “physical activity paradox,” where occupational PA may not confer the same benefits as leisure‐time PA due to differences in patterns and psychosocial context (Coenen et al. 2018; Holtermann et al. 2012). Therefore, the domain of PA (occupational vs. leisure) may be a critical modifier of its relationship with stroke risk, highlighting the need for domain‐specific assessment in future research.

This study has some strengths. First, as far as we know, this is the first prospective cohort study exploring the association between PA and the onset of stroke among Chinese adults. We used a large, nationally representative sample covering 28 provinces in mainland China. Consequently, our conclusions may be generalized to the broader population of middle‐aged and older Chinese adults. Second, this investigation employed a 9‐year observational cohort design incorporating systematic adjustment for multiple demographic, behavioral, and clinical confounders. Third, we have analyzed various physical activity patterns, such as MVPA, VPA, MVA, LPA, and TPA. Finally, subgroup analyses by common confounding variables were also performed to assess the robustness of the findings.

The observed protective association specifically with guideline‐based MVPA may be explained by its role in building a broad physiological reserve (Giustiniani and Quartarone 2024). This reserve integrates cardiometabolic, motor, and potentially cognitive capacities. Functioning together, these capacities enhance resilience against vascular injury, thereby providing a biological basis for the statistical associations observed in our cohort. Our finding aligns with the view that sustained engagement in MVPA is a key stimulus for building and maintaining such an integrative reserve. Crucially, an individual's premorbid status across these domains is a well‐established determinant of functional recovery after a stroke, as evidenced by studies linking higher premorbid function to better outcomes (van Allen et al. 2024; Nozoe et al. 2024; Wang et al. 2025). It also plausibly influences the susceptibility to the initial stroke event. This perspective may offer a unifying explanation for the significant associations observed in certain subgroups within our study, such as younger or rural participants, who might possess distinct reserve characteristics or activity patterns. Future investigations should seek to integrate objective measures of physical activity with direct assessments of cognitive and functional reserve to clarify their combined influence on stroke risk and long‐term prognosis.

There are also limitations to this study. First, both PA and stroke were ascertained via self‐report. This reliance may introduce recall bias and non‐differential misclassification, potentially biasing estimates toward the null. Second, the questionnaire lacked specific dietary questions. Consequently, we could not adjust for diet, a potential confounder, in our multivariable models. Third, we were unable to determine the precise timing of stroke onset relative to changes in physical activity, as functional status was assessed at survey waves. Fourth, the activity captured in our study was predominantly occupational. This context may limit the generalizability of our findings to populations where leisure‐time physical activity is more common. Furthermore, residual selection bias cannot be entirely excluded despite our baseline comparisons. For example, the lower prevalence of smoking in the included sample compared to excluded participants may render our estimates conservative, potentially biasing the associations toward the null. Finally, we did not perform sensitivity analyses on our PA data processing methods (e.g., imputation and truncation thresholds).

## 5 Conclusions

In summary, this prospective cohort study indicated that guideline‐based MVPA is associated with a lower risk of stroke in middle‐aged and older Chinese adults. Given that the PA in this cohort was predominantly occupation‐based and self‐reported, future research should combine objective PA measurement with direct assessments of cognitive and functional reserve to better understand the multidimensional pathways to stroke prevention.

## Author Contributions

B. Z. and L. D. conceived and designed the study; H. J. and B. Z. conducted statistical analyses and data interpretation and drafted the manuscript; and Y. C. and L. D. contributed to the data curation and revised the manuscript. B. Z. is the study guarantor. All authors have read and approved the final version of the manuscript.

## Funding

This project is funded by the National Key Clinical Specialty Construction Project (Grant No. 2023283), Tianjin Key Medical Discipline Construction Project (Grant No. TJYXZDXK‐3‐003D), and Tianjin Health Research Project (Grant No. TJWJ2025QN104).

## Ethics Statement

The Peking University institutional review board gave its approval to the CHARLS project (IRB00001052‐11015).

## Consent

Written informed consent was acquired before participation.

## Conflicts of Interest

The authors declare no conflicts of interest.

## Data Availability

The data that support the findings of this study are available in Peking University Open Research Data at http://charls.pku.edu.cn/en/index.htm.
